# Bilateral Ovarian Serous Borderline Tumor with Non-Invasive Endometrial Implants

**DOI:** 10.1155/2023/4845887

**Published:** 2023-06-08

**Authors:** Melissa Bou Malham, Jordy Mehawej, Andreas Filippaios, Christina Kushnir, Paulette Mhawech-Fauceglia

**Affiliations:** ^1^Department of Medicine, University of Florida, Gainesville, FL, USA; ^2^Department of Medicine, UMass Chan Medical School, Worcester, MA, USA; ^3^Department of Gynecologic Oncology, Women's Cancer Center of Nevada, Las Vegas, NV, USA; ^4^Department of Anatomic Pathology, Sonic Health Care, Las Vegas, NV, USA

## Abstract

Herein, we are presenting a case of a 33-year-old woman who presented to the emergency department complaining of persistent lower abdominal pain of one-day duration. Physical examination revealed abdominal tenderness with right lower quadrant rebound tenderness. Computed tomography abdomen/pelvis showed a 6 cm possible necrotic mass of the left ovary with moderate amount of complex ascites. A laparoscopic left oophorectomy with bilateral salpingectomy, right ovarian biopsy, and appendectomy were performed without complications. The cut surface of the left ovary showed a 9.7 cm × 8 cm × 4 cm ovarian mass, and the cut surface revealed multiple gray-tan friable papillary excrescence. Microscopic evaluation showed findings consistent with left and right ovarian serous borderline tumor (SBT). Subsequently, a tumor staging was conducted with total laparoscopic hysterectomy, pelvic and periaortic lymph node dissection, and omentectomy. The endometrium sections showed several small foci of SBT within the endometrial stroma, consistent with non-invasive implants of the endometrium. The omentum and lymph nodes were all negative for malignancy. SBTs associated with endometrial implants are very rare with only one case reported in the literature. Their existence can cause diagnostic challenges, and they should be acknowledged for early diagnosis and to plan for patient's treatment and outcome.

## 1. Introduction

Ovarian serous borderline tumor (SBT) is a low-grade epithelial neoplasm that share molecular alterations with low-grade serous carcinomas (LGSOC) [1]. It is the most common type of borderline ovarian tumors. SBTs are neoplasms of generally younger patients and are commonly seen in the fourth and fifth decades [2]. According to the World Health Organization, most patients present with non-specific symptoms including abdominal pain or abdominal distension [3]. Microscopically, they are subclassified into: (1) conventional SBT, which shows hierarchically branching papillae lined by stratified epithelium with mild to moderate atypia; and (2) micropapillary/cribriform SBT, which shows multiple non-branching filiform structures without fibrovascular cores that are five times longer than they are wide, originating directly from the bulbous central [4]. The hallmark of SBT is the lack of stromal invasion. Therefore, ample sampling is needed to exclude serous carcinoma. They have a favorable prognosis when diagnosed at an early stage [5]. Owing to their borderline malignant potential, peritoneal implants and/or lymph node involvement can occur at higher stages. Extra-ovarian disease is more commonly seen in the omentum, ipsilateral ovarian surface, peritoneal surface of organs, and abdominal wall, [6] leaving endometrial implant as extremely rare occurrences with only one case reported in the literature.

We report a case of a 33-year-old woman diagnosed with bilateral ovarian SBT with a non-invasive endometrial implant. We review the relevant literature and its application to diagnosis, prognosis, and treatment.

## 2. Case Report

A 33-year-old woman G2P2, presented to the emergency department for persistent lower abdominal/pelvic pain of one-day duration. On physical examination, the patient had abdominal pain with right lower quadrant mild rebound tenderness. The patient reported no family history of cancer. Pelvic and transvaginal ultrasound showed a large complex mixed cystic and solid left adnexal lesion suspicious for ovarian adnexal neoplasm; and a normal appearing right ovary. Complex free fluid in the pelvis was also noted. Subsequently, computed tomography abdomen/pelvis with intravenous contrast showed a 6 cm necrotic pelvic mass and a moderate amount of ascites. These findings warranted the patient's admission for further gynecologic evaluation. The patient was scheduled for a laparoscopic left oophorectomy with bilateral salpingectomy, right ovarian biopsy, and appendectomy.

**Figure 1:**
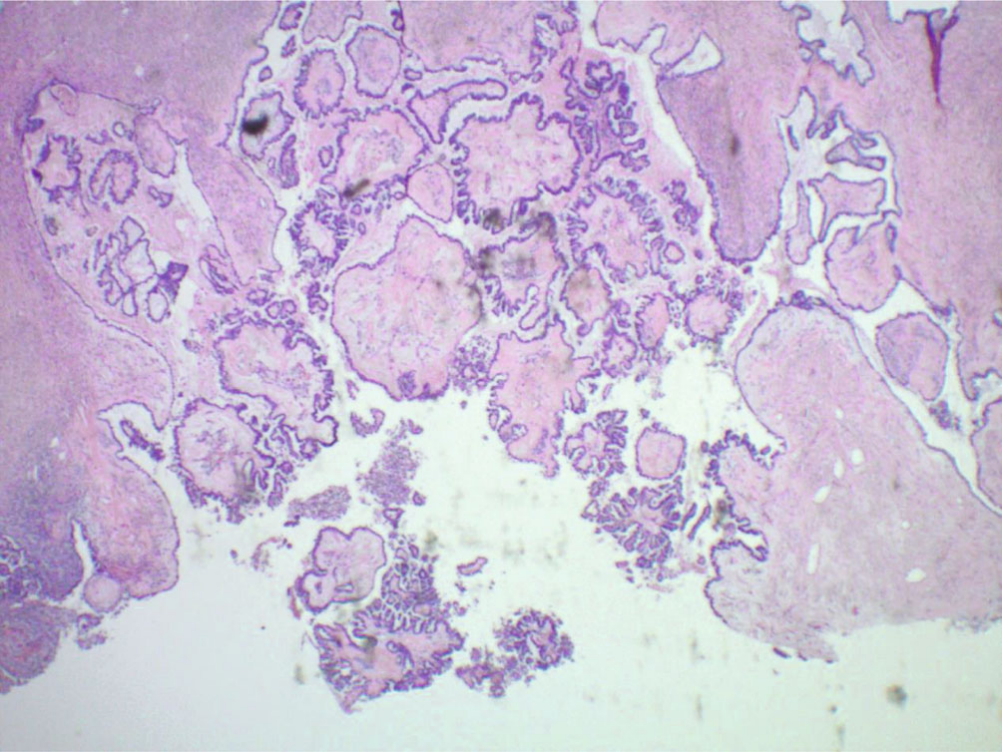
Sections of the right ovary show tumor with papillary lesions. These papillae have fibrous stalk, and they are lined by pseudostratified cuboidal epithelium. No stromal invasion seen (magnification: ×10).

**Figure 2:**
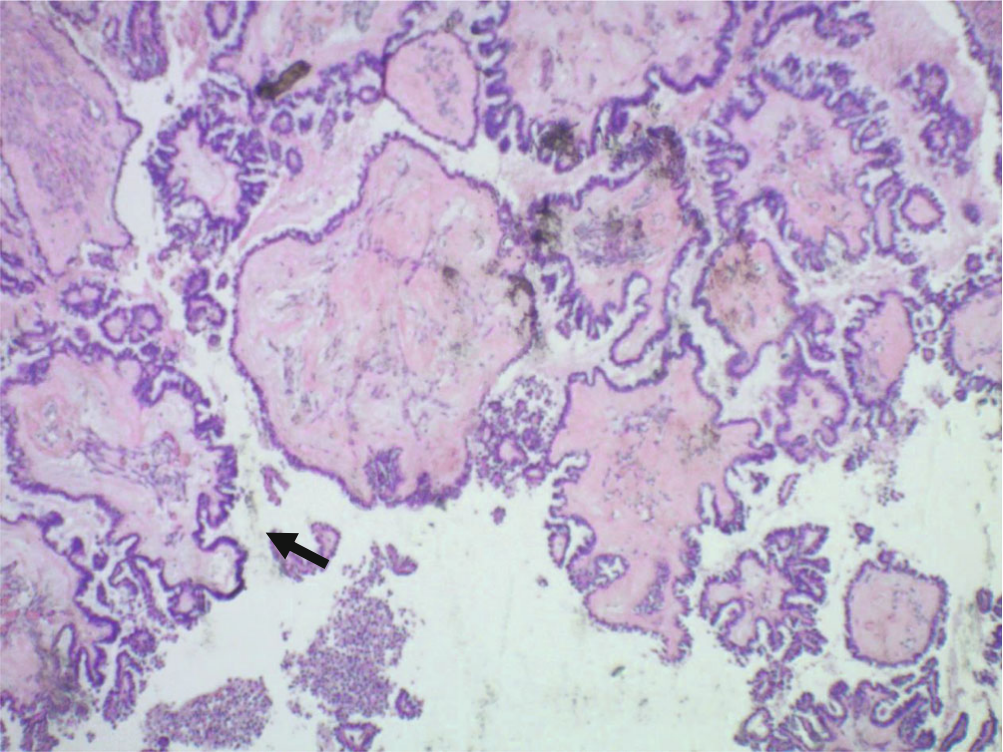
Higher magnification shows epithelia tufting (black arrow; magnification: ×20).

Gross examination of the left ovary showed multiple fragmented and disrupted fragments measuring 9.7 cm × 8 cm × 4 cm in aggregates. The inner linings displayed multiple gray-tan friable papillary excrescences, up to 2.3 cm in greatest dimension. No discrete uninvolved ovarian parenchyma was identified, and the wall averaged 0.3 cm thick. The right ovarian biopsy showed a 0.7 cm tan portion of soft tissue. On microscopic examination, the left ovary was replaced by tumor characterized by papillary structures lined by pseudostratified epithelium with low to moderate atypia. Similar morphology was seen on the right ovary and pelvic washing (Figures 1 and 2). The final pathologic diagnosis was SBT of the left and right ovaries with positive pelvic washing. Tumor was staged as at least stage IC3 (pending complete staging).

**Figure 3:**
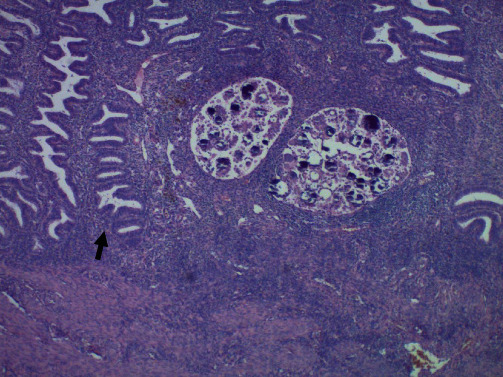
Sections of the endometrium shows benign proliferative endometrial glands (black arrow). In the endometrial stroma, nests of tumor are present (magnification: ×10).

**Figure 4:**
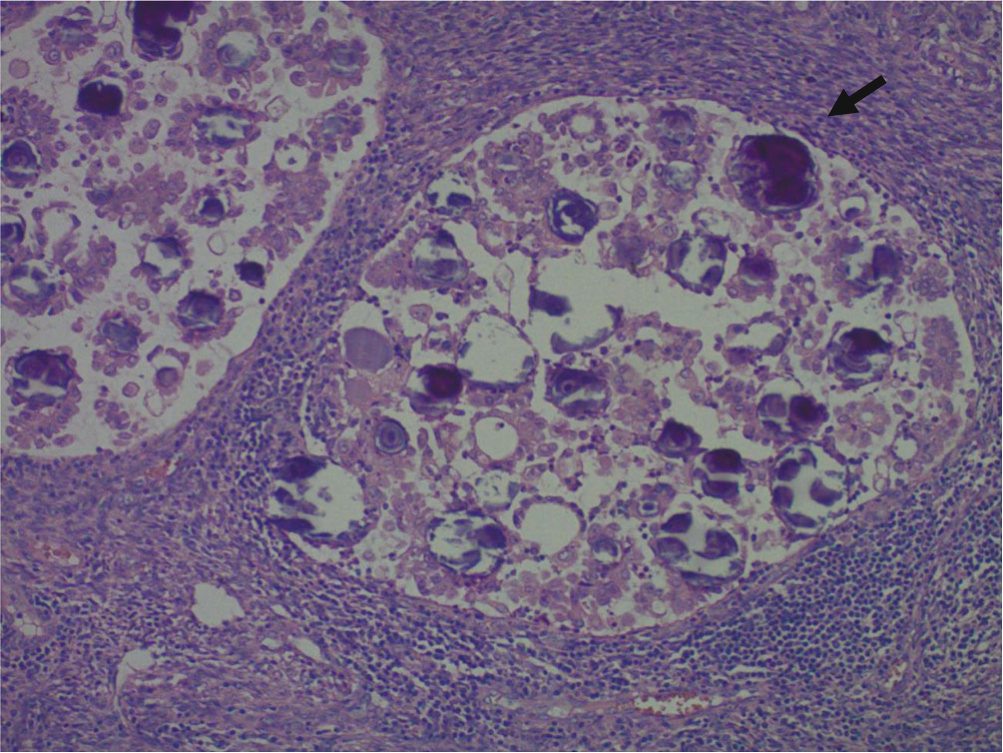
Higher magnification shows tumor with papillary architecture and numerous psammoma bodies (black arrow) similar to those seen in the right and left ovary (magnification: ×20).

Subsequently, a total laparoscopic hysterectomy, pelvic and periaortic lymph node dissection, and omentectomy for complete staging were done. The surgery was uneventful. The gross examination was unremarkable except that sectioning of the right ovary revealed multiple smooth-walled fluid-filled cysts up to 1.0 cm. Within the larger cyst was a 0.9 cm firm gray-white area of papillary excrescences (Figures 3 and 4). The surgical specimens were frozen. Microscopically, the right ovary showed a complex ovarian cyst showing a SBT correlating with the previous right ovary biopsy. The endometrium showed several tiny foci of SBT (all less than 1 mm) within the endometrial mucosa, consistent with non-invasive implants of the endometrium. The omentum and pelvic and aortic lymph nodes were all negative for malignancy. Based on the new findings, the American Joint Committee on Cancer and International Federation of Gynecology and Obstetrics staging were revised to T2A and IIA, respectively [7, 8]. The patient underwent full recovery.

## 3. Discussion

About a third of patients with SBT are asymptomatic; otherwise, they might present with non-specific pelvic/abdominal pain, due to compression effect on adjacent organs and uncommonly as abdominal bloating, ascites, and distention. Prognosis is largely dependent on tumor stage, meaning patients with early stage have the same outcome as the general population, and patients with advanced stage are often associated with lower survival rate. In addition, other prognostic factors, such as invasive versus non-invasive implants as well as micropapillary pattern, have a significant effect on a patient's outcomes. Extra-ovarian implants are more commonly found in contralateral ovarian surface, omentum, diaphragm, abdominal wall, and serosal surface of other abdominopelvic organs. Uterine implants are usually seen in the serosal surface, and implants in the endometrial lining are extremely rare with only one case reported in the English literature [9]. This case was reported by Gonzalez et al. in a 31-year-old woman. Similarly, our case here, occurs in a 33-year-old. Gonzalez et al. reported a unilateral SBT as opposed to our bilateral case of SBT. Furthermore, the endometrial implant reported by Gonzalez et al. was discovered after the patient presented with irregular vaginal bleeding 3 months post-ovarian surgery, and endometrial biopsy showed simple hyperplasia without atypia. However, a follow-up endometrial biopsy at 11 months revealed serous cells with similar patterns of immunohistochemistry staining as tissue from the primary ovarian tumor. In our case, endometrial implant was identified, after complete surgical staging using total laparoscopic hysterectomy and post-SBT diagnosis in both ovaries. This emphasizes the importance of complete staging after SBTs are diagnosed. However, hysterectomy is not usually done in tumor staging at this age group due to fertility and pregnancy desire. However, endometrial sampling is warranted. In addition, that might underestimate its occurrence in the endometrium as the uterus will be kept in situ. Extra-ovarian implants are present in 30–40% of patients with SBTs and up to 30–40% of those patients die of the disease [3]. Implants and their subtypes (invasive and non-invasive) are important prognostic indicators for SBTs. The invasive pattern most commonly occurs in patients with the micropapillary type of SBT and tend to have an adverse prognosis [10]. As such, proper classification of the tumor and implants are required for adequate diagnosis of SBTs. It is important to note that SBTs are different from LGSOC and have different treatments. In fact, a study hypothesized that SBT might serve as a precursor of LGSOC due to a diagnosis shift from LGSOC to SBT in a time-specific manner. However, this is still subject to debate. This emphasizes the importance of accurate diagnosis to prevent over- or under-treatment of such tumors [11].

As for the pathogenesis of implants in the endometrium from ovarian SBT, we can extrapolate from the implants to other extra-ovarian sites, where tumor cells have the ability to shed malignant cells into the peritoneum; thereby, giving rise to peritoneal implants. We also agree with Gonzalez et al.'s hypothesis that tumor cells might detach from the ovarian tumor and migrate to the endometrial cavity though the fallopian tubes, thus, attempting to actively implant in the endometrium. Since this finding in the endometrium is very focal, it can easily be missed on endometrial biopsy.

Finally, making the diagnosis of SBT implant on endometrial biopsy can create diagnostic challenges in the absence of history or clinical data. These foci can be misinterpreted as serous carcinoma of the endometrium. However, patient's age, clinical findings of ovarian mass, and presence of ascites can lead to the right diagnosis.

The recommended treatment for SBT is hysterectomy and bilateral salpingo-oophorectomy with complete staging [12]. There is great debate regarding the benefit of complete staging when patients present with unilateral tumors because only 15% of such tumors are associated with extra-ovarian disease. Bilateral SBTs are associated with extra-ovarian disease in 56% of cases and warrant complete staging [13]. A fertility-preserving surgery is therefore an important consideration in women of reproductive age. Preserving the uterus and at least one ovary should be discussed with young patients despite this treatment having a higher recurrence rate compared with radical treatment (10–20% vs. ~5% for radical surgery) [14]. A study assessing the outcomes of 65 patients with SBTs undergoing conservative surgery showed that spontaneous pregnancy can be achieved at the expense of a high recurrence rate (58% of cases recurred) and 3 deaths occurred [15]. Overall, recurrence rates are between 3% and 10% [16] A systematic review showed that 37% of recurrences occur during the first 2 years, 32% relapse more than 5 years after diagnosis, and 10% occur more than 10 years after diagnosis [17]. These numbers raise the question of whether these tumors are relapses or have developed de novo. Although complete surgical staging was done in our case, and prolonged follow-up is advised due to the risk of recurrence and occasional transformation to invasive carcinoma. The requirement of pelvic and paraaortic lymphadenectomy has been controversial because recurrence and survival rates for patients with spared and unspared lymph nodes were similar [18]. There is yet no proven benefit from chemotherapy or radiotherapy even in cases of advanced disease [19].

In summary, our case is unique since it is the second case to report an ovarian SBT associated with non-invasive uterine implants, and the only case to report a bilateral ovarian SBT with non-invasive endometrial implants. SBTs associated with uterine implants should be acknowledged, and early diagnosis is crucial for treatment and improved patient outcome.

## References

[1] Hauptmann S., Friedrich K., Redline R., Avril S. (2017). Ovarian borderline tumors in the 2014 WHO classification: evolving concepts and diagnostic criteria. *Virchows Archiv*.

[2] Veran-Taguibao S., Taguibao R. A. A., Gallegos N. (2019). A rare case of ovarian serous borderline tumor with brain metastasis. *Case Reports in Pathology*.

[3] Srinivasamurthy B. C., Kulandai Velu A. R., Krishnan N., Patil A. S. (2015). Ovarian serous borderline tumors with noninvasive and invasive peritoneal implants: a case report each. *Journal of Cancer Research and Therapeutics*.

[4] Bell D. A., Longacre T. A., Prat J. (2004). Serous borderline (low malignant potential, atypical proliferative) ovarian tumors: workshop perspectives. *Human Pathology*.

[5] Rasmussen E. L. K., Hannibal C. G., Dehlendorff C. (2017). Parity, infertility, oral contraceptives, and hormone replacement therapy and the risk of ovarian serous borderline tumors: a nationwide case-control study. *Gynecologic Oncology*.

[6] Sharma A., Lastra R. R. https://www.pathologyoutlines.com/topic/ovarytumorserousborderline.html.

[7] Berek J. S., Renz M., Kehoe S., Kumar L., Friedlander M. (2021). Cancer of the ovary, fallopian tube, and peritoneum: 2021 update. *International Journal of Gynaecology and Obstetrics*.

[8] Amin M. B., Greene F. L., Edge S. B. (2017). The eighth edition AJCC cancer staging manual: continuing to build a bridge from a population-based to a more “personalized” approach to cancer staging. *CA: A Cancer Journal for Clinicians*.

[9] Gonzalez R. S., Chamberlain B. K., Giannico G., Fadare O., Crispens M. A. (2013). Ovarian serous borderline tumor identified in endometrial biopsy: a case report. *Gynécologie et Obstétrique*.

[10] Minagawa M., Maeda M., Shimauchi M., Kishi H., Teshima S. (2018). Serous borderline tumor with micropapillary pattern of the right ovary that developed 6 recurrences over 30 years after primary surgery. *Gynecologic Oncology Reports*.

[11] Matsuo K., Machida H., Grubbs B. H. (2020). Diagnosis-shift between low-grade serous ovarian cancer and serous borderline ovarian tumor: a population-based study. *Gynecologic Oncology*.

[12] Hacker K. E., Uppal S., Johnston C. (2016). Principles of treatment for borderline, micropapillary serous, and low-grade ovarian cancer. *Journal of the National Comprehensive Cancer Network*.

[13] Seidman J., Kurman R. (2008). The global library of women’s medicine (ISSN: 1756-2228).

[14] du Bois A., Trillsch F., Mahner S., Heitz F., Harter P. (2016). Management of borderline ovarian tumors. *Annals of Oncology*.

[15] Gouy S., Maria S., Faron M. (2021). Results after conservative surgery of stage II/III serous borderline ovarian tumors. *Annals of Surgical Oncology*.

[16] Lenhard M. S., Mitterer S., Kümper C. (2009). Long-term follow-up after ovarian borderline tumor: relapse and survival in a large patient cohort. *European Journal of Obstetrics, Gynecology, and Reproductive Biology*.

[17] du Bois A., Ewald-Riegler N., de Gregorio N. (2013). Borderline tumours of the ovary: a cohort study of the Arbeitsgemeinschaft Gynakologische Onkologie (AGO) Study Group. *European Journal of Cancer*.

[18] Seidman J. D., Kurman R. J. (2000). Ovarian serous borderline tumors: a critical review of the literature with emphasis on prognostic indicators. *Human Pathology*.

[19] Seong S. J., Kim D. H., Kim M. K., Song T. (2015). Controversies in borderline ovarian tumors. *Journal of Gynecologic Oncology*.

